# Insight into dual fluorescence effects induced by molecular aggregation occurring in membrane model systems containing 1,3,4-thiadiazole derivatives

**DOI:** 10.1007/s00249-021-01569-7

**Published:** 2021-09-13

**Authors:** Melinda David, Iwona Budziak-Wieczorek, Dariusz Karcz, Monica Florescu, Arkadiusz Matwijczuk

**Affiliations:** 1 Faculty of Medicine, Transilvania University of Brașov, 500019 Brașov, Romania; 2 Department of Chemistry, University of Life Sciences in Lublin, 20-950 Lublin, Poland; 3 Department of Chemical Technology and Environmental Analytics (C1), Faculty of Chemical Engineering and Technology, Cracow University of Technology, Warszawska 24, 31-155 Kraków, Poland; 4 Department of Biophysics, University of Life Sciences in Lublin, Lublin, Poland

**Keywords:** 1,3,4-Thiadiazole, Molecular spectroscopy, SPR and EIS, Molecular aggregation and ESIPT, Dual fluorescence effects, Liposome systems

## Abstract

This work reports on biophysical insights into the excited state intramolecular proton transfer (ESIPT) processes taking place in three 1,3,4-thiadiazole derivatives that served as model compounds, on which electronic absorption, fluorescence, Fourier-transform infrared spectroscopy (FTIR), surface plasmon resonance (SPR) and electrochemical impedance spectroscopy (EIS) studies were performed. The fluorescence spectra recorded in various solvents revealed an interesting dual fluorescence effect. In molecules in their monomeric form, the effect is associated with the ESIPT phenomenon, and may be further enhanced by aggregation-related effects, such as aggregation-induced emissions. Other spectroscopic studies on the selected molecules in a liposomal medium as a model revealed that, in a biomimetic environment, they can exist in both monomeric and aggregated forms. In both cases, however, the effects observed are closely related to the lipid’s main phase transition temperature and the structure of the molecule. Introduction of specific substituents to the phenyl moiety either allows or prevents proton transfer from occurring in the excited state. The hydrophobicity changes in a lipid environment may result in an emergence of specific molecular forms and therefore either facilitate or hinder ESIPT processes. SPR and EIS confirmed the significant hydrophobicity changes in the model lipid systems, while FTIR measurements revealed a notable influence of 1,3,4*-*thiadiazoles on the fluidity of liposomal membranes. The results obtained clearly show that the thiadiazole derivatives are very good model molecules for studying hydrophobic-hydrophilic environments, and particularly with polymers or liposomes used as drug delivery systems.

**Supplementary Information:**

The online version contains supplementary material available at 10.1007/s00249-021-01569-7.

## Introduction

Thiadiazoles, and particularly the series of 1,3,4-thiadiazole derivatives studied by our group (Budziak et al. 2019; Czernel et al. 2018; Matwijczuk et al. 2017, 2018), demonstrate a wide spectrum of pharmacological effects, including anticancer (Gomha et al. 2015), neuroprotective (Sarafroz et al. 2019), antibacterial (Zoumpoulakis et al. 2012), antioxidative (Hamama et al. 2013), and antimycotic properties (Karcz et al. 2020), as well as other effects (Gür 2019; Serban et al. 2018). Various 1,3,4-thiadiazoles often demonstrate non-typical physiochemical properties, such as the dual fluorescence emission, crystal polymorphism/solvatomorphism, numerous interactions occurring in model lipid systems, and synergistic antimicrobial effects with commercial antibiotics(Czernel et al. 2018; Hoser et al. 2013, 2018; Karcz et al. 2017, 2020).

Given the high biological activity of 1,3,4-thiadiazoles, their potential practical applications in modern medicine must be preceded by thorough spectroscopic characterization (Balan et al. 2019; Shahzad et al. 2001, 2009), especially with use of the fluorescence studies (Matwijczuk et al. 2017). In this context, the ability of 1,3,4-thiadiazoles to generate the so-called dual fluorescence effect is particularly worth investigating (Budziak et al. 2019). The literature provides a number of possible explanations for this effect, among which the intermolecular charge transfer (CT) inducing the emergence of specific CT states, often involving twisting of the molecule (twisted intramolecular charge transfer—TICT), is most commonly reported (Hu et al. 2009; Li et al. 2019). The alternative explanations of dual fluorescence effects refer to the formation of excimer structures (Chen et al. 2018), breaking Kasha’s rule (Brancato et al. 2015; Younes and Zhu 2012), or processes strictly related to molecular aggregation (Matwijczuk et al. 2017, 2018). However the most interesting explanation for dual fluorescence highlights processes of multistage photo-tautomerization, i.e. ESIPT (excited-state intramolecular proton transfer) (Bhattacharyya et al. 2020; Cheng et al. 2015; Hao et al. 2019; Heyer et al. 2017; Suzuki et al. 2014), which seems to be the most accurate model (Massue et al. 2018) for the molecules under discussion. In recent years, a number of reports discussing aggregation-induced emissions (AIE) were published, suggesting a combination of ESIPT and AIE as jointly responsible for the emergence and often significant enhancement of dual fluorescence. Compounds which can change their fluorescence emission dependent on their immediate environment tend to constitute excellent fluorescence probes. Fluorophores undergoing an overlap of ESIPT and AIE effects are one such example (Dai et al. 2020). This effect is often characterised by keto-enol or imino-enamino tautomerism.

The 1,3,4-thiadiazoles and especially those which demonstrate the ESIPT-related fluorescence are of interest mainly due to their potential practical application in cellular bio-imaging. Furthermore, spectroscopic studies of the dual fluorescence phenomenon occurring in various solvents, aqueous media, or systems with nano-structural additives have recently gained considerable attention and were performed as complementary experiments for this work. Nonetheless, to date, there have not been any reports on the analysis of dual fluorescence effects in model biological systems (e.g. liposomal ones).

**Scheme 1.**
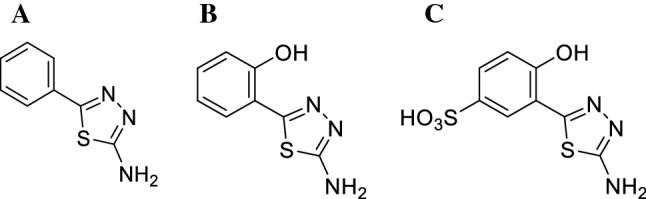
Chemical structure of compounds: benzoic thiadiazole (TB), salicylic thiadiazole (TS) and sulfosalicylic thiadiazole (TSF)

Therefore, the main novelty and objective of our current study was to carry out a detailed spectroscopic analysis of the selected 2-amino-1,3,4-thiadiazoles, namely *2-amino-5-phenyl-1,3,4-thiadiazole* (**TB**), *2-amino-5-(2-hydroxyphenyl)-1,3,4-thiadiazole* (**TS**), and *2-amino-5-(2-hydroxy-5-sulfobenzoyl)-1,3,4-thiadiazole* (**TSF**) (Scheme 1) in liposomal systems obtained with the use of phospholipid dipalmitoylphosphatidylcholine (DPPC). For this purpose, the absorption and electronic fluorescence spectra were recorded, including measurements of emission, excitation and resonance light scattering (RLS), and Fourier-transform infrared spectroscopy (FTIR) spectra. The FTIR spectroscopy measurements confirmed that the molecules of the studied thiadiazoles interact more strongly with the hydrophilic part of the liposomal membrane. The above studies were followed by measurements conducted in liposomal systems with use of surface plasmon resonance (SPR) and electrochemical impedance spectroscopy (EIS). These revealed a correlation between changes in the model liposomal membrane’s fluidity and the prevalence of either monomeric or aggregated molecules, as corroborated by fluorescence measurements. SPR measurements allowed for detailed description of the biomolecular interactions occurring in the membrane model system with the addition of selected thiadiazoles, together with changes in membrane fluidity. In turn, EIS measurements allowed investigation of the impact of the effect of temperature on the model liposomal membrane and more precise characterization of compound interactions with the membrane in the most characteristic temperature range, related to phase transitions in the DPPC lipids.

The research presented in this article shows that the selected molecules from the 1,3,4-thiadiazole group can be a source of new molecular probes, which are consistently wanted in molecular biology and related sciences (Matwijczuk et al. 2017; Mohapatra and Mishra 2012; Starzak et al. 2019).

## Materials and methods

### Materials

2-amino-5-phenyl-1,3,4-thiadiazole (TB), 2-amino-5-(2-hydroxyphenyl)-1,3,4-thiadiazole (TS) and 2-amino-5-(2-hydroxy-5-sulfobenzoyl)-1,3,4-thiadiazole (TSF) were synthesised according to the previously reported protocol (Budziak et al. 2019). The phospholipid dipalmitoylphosphatidylcholine (DPPC) with > 99% purity was purchased from Avanti Polar Lipids. All solvents used were purchased from Sigma-Aldrich. Phosphate-buffered saline (PBS) was purchased from Sigma-Aldrich.

The TB, TS, and TSF (about 1 mg) samples were dissolved in 2 mL of solvent (ethanol, butan-1-ol, chloroform and toluene). The absorbance intensity was adjusted by the addition of an appropriate aliquot of TB, TS, and TSF into 2 mL of solvent. The solubility of TB, TS, and TSF in solvents used was limited, which is why the exact concentrations of samples remained undetermined.

## Methods

### Formation of liposomes

Multilamellar liposomal formulations were prepared using the thin-film hydration method. First, the lipids were dissolved in chloroform at a concentration of 5 × 10^−2^ M. and TS and TSF were dissolved at a concentration 1 × 10^−3^ M (stock solution). Liposome formation with TB, TS and TSF (5, 10, 15 and 20 molar % relative to the lipid) was carried out in glass tubes. By mixing corresponding volume ratios of chloroform solution with the lipid and methanol solution with thiadiazoles the required concentrations were obtained. The mixture was evaporated under a dry nitrogen stream. Then, the samples were conditioned under vacuum for about 1 h to form a thin, homogeneous, solvent-free film. A PBS buffer with pH 7.4 was added to the dry sample to obtain a concentration of 0.37 mg DPPC/1 ml buffer (5 × 10^−4^ M). Samples were placed in a water bath at 45 °C for 5 min. Next, they were removed and shaken in a vortex mixer for about 10 s before being placed in the water bath to initiate the hydration process (Kluczyk et al. 2016; Varona et al. 2011). The entire process was repeated 3 times. At the end, samples were put into an ultrasonic bath for 15 min (45 °C). Prior to the experiment, the mixtures were stored at 4 °C for 24 h before the measurement to ensure complete lipid hydration and complete equilibration of the liposomal system (Amin et al. 2018; Budziak et al. 2018; El-Nesr et al. 2010; Franze et al. 2020; Moreno et al. 2009).

### Measurements of electronic absorption and fluorescence spectra

The electronic absorption spectra of TB, TS and TSF were recorded using a double-beam UV–Vis spectrophotometer Cary 300 Bio Varian (Middelburg, The Netherlands) equipped with a thermostatted cuvette holder with a 6 × 6 multicell Peltier block. The temperature was controlled with a thermocouple probe (Cary Series II from Varian) placed directly in the sample.

A Cary Eclipse spectrofluorometer (Varian) was used to record fluorescence excitation, emission, and synchronous spectra. All the fluorescence spectra were recorded at 0.5 nm resolution with due consideration for the lamp and photomultiplier tube spectral characteristics. Resonance light scattering (RLS) measurements were carried out by synchronously scanning both the excitation and emission monochromators (there was no interval between excitation and emission wavelengths) at the spectral resolution of 5 nm. Grams/AI 8.0 software (Thermo Electron Corporation; Waltham, Massachusetts, United States) was used to analyse the recorded data.

### FTIR measurements

Measurements of ATR-FTIR background-corrected spectra (25 scans for each sample) were carried out with the use of a HATR Ge trough (45° cut, yielding 10 internal reflections) crystal plate at 20 °C, and were recorded with a 670-IR spectrometer (Agilent, USA). The Ge crystal was cleaned with ultra-pure organic solvents (Sigma-Aldrich). The instrument was continuously purged with argon for 40 min. before and during measurements. Absorption spectra at a resolution of one data point per 1 cm^−1^ were obtained in the region between 4000 and 400 cm^−1^. Scans were Fourier-transformed and averaged with Grams/AI 8.0 software (Thermo Fisher Scientific, USA). Dry DPPC/thiadiazoles films were prepared by evaporating the mixture under a stream of nitrogen.

### SPR measurements

The SPR analyser system, MP-SPR NaviTM 200 OTSO, and planar gold SPR chips (BioNavis LTD, Finland) with the corresponding software packages for data acquisition and analysis (SPR Navi Control, SPR Navi Data Viewer) were used. The variation in SPR parameters which occur between the model DPPC liposomal membrane system and the analogues (TB, TS and TSF) were evaluated as a function of temperature. The SPR analyser was based on the Kretschmann configuration in which a polarized, collimated light beam undergoes total internal reflection at a glass/metal/dielectric interface. The system is equipped with 2 lasers with a wavelength of 670 nm. The SPR chips are formed by a glass substrate coated with a 50 nm thin gold layer which produces the SPR effect, and were used as such. The SPR system has a thermostat and the working temperature was varied from 30 to 43 °C. All measurements were performed in a 100 µL electrochemical-SPR cell, the samples were injected manually and temperature dependent experiments were carried out.

### EIS measurements

The SPR system was equipped with an 100 µL electrochemical-SPR cell, which was used for electrochemical measurements and connected to the electrochemical interface PalmSens3 (Palm Instruments BV, The Netherlands) controlled by PSTrace 5.8 software. The gold SPR chip was used as a working electrode, together with a platinum wire as counter electrode and a silver wire as reference electrode. The surface of the gold chip was 1 cm^2^, and this value was used to normalize the results. The working temperature was varied between 30 and 43 °C, using the SPR system thermostat. An rms perturbation of 10 mV was applied over the frequency range 0.05–30,000.00 Hz, with 8.5 frequency values per frequency decade. The obtained spectra were recorded at a potential of − 0.35 V vs. Ag, and plotted in the form of complex plane diagrams—Nyquist plots. Based upon the principles of EIS and using the PSTrace 5.8 software with FRA module, the equivalent electrical circuit best fitting the experimental data with corresponding electrical parameters were inferred.

### Fluorescence quantum yield

The fluorescence quantum yields of TB, TS and TSF solutions were determined using 7-diethylamino-4-methylcoumarin (coumarin1) as the fluorescence standard. The measurements were carried out in ethanol $\phi_{{\text{R}}}$= 0.73 (Jones et al. 1985). The final fluorescence quantum yield values were calculated based on Formula (1),

1 $$ \Phi_{F\left( X \right)} = \Phi_{{R\left( {EtOH} \right)}} \left( {\frac{{A_{{R\left( {EtOH} \right)}} }}{{A_{X} }}} \right)\left( {\frac{{I_{X} }}{{I_{{R\left( {EtOH} \right)}} }}} \right)\left( {\frac{{\eta_{X}^{2} }}{{\eta_{{R\left( {EtOH} \right)}}^{2} }}} \right) $$

where the subscript *X* denotes the corresponding TB, TS or TSF compounds in different solvents, *A* is the value of absorbance at the excitation wavelength, *I*—the area under the emission curve, and η the refractive index of the solv. *R*—coumarin1.

## Results and discussion

The 1,3,4*-*thiadiazole model compounds **TB**, **TS**, and **TSF** were selected in order to provide the structural differences in their respective resorcinol systems (Scheme 1A–C). In more detail, the **TB** analogue possesses a bare (unsubstituted) phenyl ring, while there is an -OH group present in **TS**, and both –OH and -sulfo moieties present in **TSF.** The –OH groups in both **TS** and **TSF** reside in the *ortho* position related to the thiadiazole ring, while the HO_3_S– group in **TSF** is attached to the *meta* position. Finally, each thiadiazole derivative has an -NH_2_ group attached to the position 2 of the 1,3,4-thiadiazole ring. The selection of model compounds was made based on the possibility for hydrogen bonding between the –OH groups and the neighbouring thiadiazole nitrogen (**TS**, and **TSF**).

The presence of substituents additionally enable the possibility for various intermolecular interactions leading to the formation of aggregates with other neighbouring molecules (both 1,3,4-thiadiazoles and solvent molecules).

### Studies of spectroscopic effects in organic solvents

**Fig. 1:**
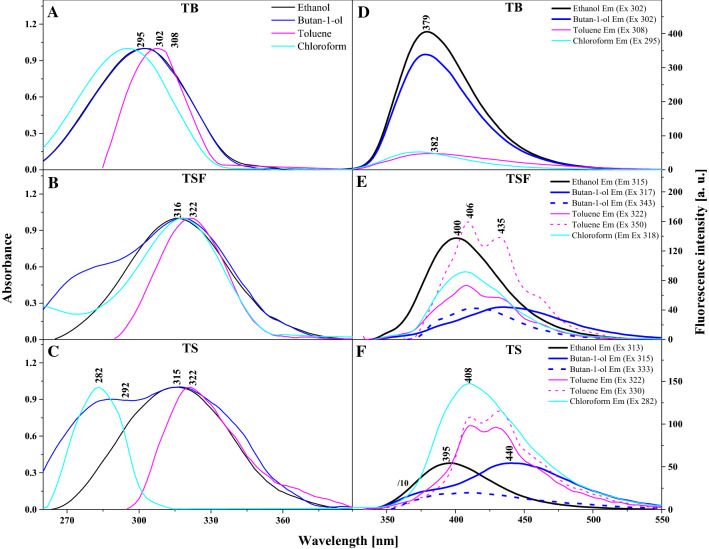
Electron absorption spectra of thiadiazoles dissolved in ethanol, butan-1-ol, toluene and chloroform. **A** TB, **B** TSF, **C** TS. The spectra were normalized at the absorbance maximum. Fluorescence emission spectra of TB (**D**), TSF (**E**) and TS (**F**) dissolved in appropriate solvents corresponding to **A**, **B**, **C**. The excitation was set at the absorbance maximum of each sample, respectively, as pointed in the figure. The spectra were measured at room temperature

Panels A, B, and C in Fig. 1 present the electronic absorption spectra for the compounds selected for the study: TB (Panel A), TSF (Panel B), and TS (Panel C) recorded in a number of solvents, both polar (Ethanol, Butan-1-ol) and non-polar (Toluene, Chloroform). Exact data reflecting the positions of the absorption maxima, fluorescence emission, Stokes shift, light refraction index *n*, and dielectric constant ε for selected solvents are provided in Table S1 (in the Supplementary Materials—SM). We can clearly see that with increasing solvent polarity, there is only a slight (by a few nm) bathochromic shift observed in the absorption spectra of all the analysed compounds. For TB Δ*λ* = 11 nm (1203 cm^−1^), for TSF Δ*λ* = 6 nm (590 cm^−1^), and for TS Δ*λ* = 7 nm (690 cm^−1^) (Fig. 1A–C and Table S1 in the SM). As seen above, for all three compounds we observe noticeable but light solvatochromic shifts of the absorption maxima, highlighting the molecules’ sensitivity towards the medium used. In all the absorption spectra of TB, TSF, and TS presented, the registered wide spectra are related to the S_0_ → S_1_ transition in the range from approximately 240 nm to 370 nm. With the maxima at approx. 300 nm for TB and 320 nm for TSF and TS, the spectra are characteristic of the *π* → π* electronic transition (Heyer et al. 2017; Rodembusch et al. 2007). It would be prudent to mention that the positions of absorption spectra in the analysed analogues were found to have changed, albeit slightly—only by a few nanometres, after drastic changes to medium polarity (Heyer et al. 2017). Thus, as confirmed by studies already available in the wider literature, these changes suggest processes related to proton transfer in the excited state to this type of, and similar molecules (Bhattacharyya et al. 2020; Rodembusch et al. 2007). Additionally, in the case of TSF and TS we can also notice, for most of the solvents used, the emergence of bands with significantly lower absorbance on the longwave side, with their maximum at ~ 360 nm. The bands are significantly less intensive than the ones discussed above, which, combined with the corresponding Stokes shift, evidences the possible presence of aggregated forms of the analogues in the analysed samples (e.g. dimers or N-aggregates, Fig. 1B, C). According to M. Kasha's theory of exciton splitting, these bands, especially on the long-wave side of the absorption spectrum, can be combined with the type of aggregation, so-called *head to tail* (Kasha et al. 1965). The aggregation capacity of molecules such as TSF or TS is also dependent on proper selection of substituent systems.

Even more interesting effects were observed during the subsequent stage of the study for TB, TSF, and TS, as presented in Fig. 1D, E, and F. These show the fluorescence emission spectra corresponding to the measurements of the absorption spectra in Fig. 1A–C. It becomes immediately apparent that relative to the ground-state spectroscopic properties of the analysed 1,3,4-thiadiazoles, the spectroscopic characteristics of their excited states are particularly interesting. In the fluorescence emission spectra of TB shown in Panel D, we can notice not only changes in intensity but also slight spectral shifts. The significant decrease in emission intensity for this analogue may be related to some intermolecular aggregation activity taking place via the -NH_2_ substituent. Changes that are even more interesting are observed for TSF and TS molecules in Panels E and F. The presented fluorescence emission spectra indicate that in these analogues, depending on the solvent used, a very intriguing and unusual effect of dual fluorescence emerges. In Panel E (TSF) we can see only a single fluorescence emission band with the maximum at approx. 406 nm present in ethanol. Bands with the maxima at approx. 410 nm and 335 nm as well as a very longwave band with the maximum at approx. 460 nm are observed in toluene after shortwave excitation, in the absorption spectra. With longwave excitation corresponding to the band of aggregation, the same maxima appear in the emission spectrum, however, this time one of the bands is significantly less intensive, which corroborates the contribution of aggregation effects. In turn, the TSF emission spectra in butan-1-ol show a more typical effect of dual fluorescence with the maxima at 395 and 440 nm.

Analogously to the case of toluene, if excitation is provided at the wavelength corresponding to the aggregation effects recorded for the absorption spectrum, the emission spectrum yields a significantly lower intensity of the observed bands. The effect is considerably more apparent for TS in Panel E—similarly to the case above, only a single fluorescence emission spectrum is observed in ethanol. In toluene, the effects were analogous to those recorded for TSF, and in butan-1-ol, shortwave excitation yielded a very wide, unstructured emission band. Meanwhile, after longwave excitation related to the aggregation capacity of this analogue, we observed a very clear effect of dual fluorescence with the maxima at approx. 370 and 440 nm. It is noteworthy that whenever the effect of dual fluorescence is observed, be it in TSF or TS, the bands are simultaneously characterised by lower intensity.

**Scheme 2.**
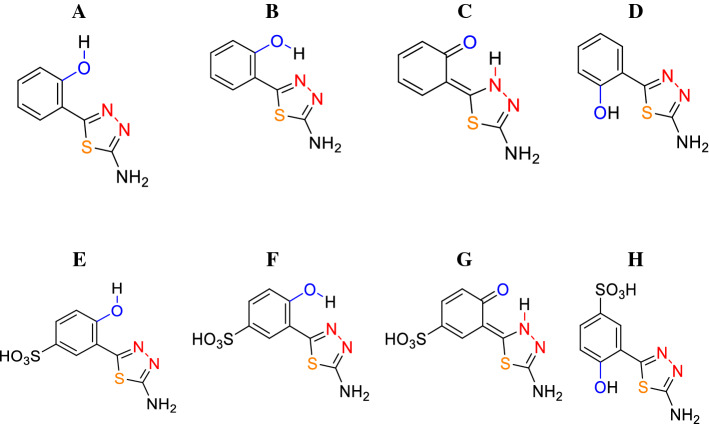
Possible conformations and ketone tautomer in salicylic thiadiazole TS (**A**–**D**) and sulfosalicylic thiadiazole TSF (**E**–**H**)

According to the literature and our earlier studies on similar effects observed in other analogues from groups including 1,3,4-thiadiazoles and 1,2,4-triazoles, changes observed in fluorescence emission spectra in response to changes in medium polarity should be associated with the ESIPT process. Longwave emission in non-polar media is often observed in structurally similar molecules capable of forming an intramolecular hydrogen bond between the proton donor and acceptor (Scheme S1A and B). The emission is related to the formation of a ketone tautomer during the excited state intramolecular proton transfer (ESIPT) process (Scheme 2C, G). The increase in solvent polarity is commonly accompanied by a hypsochromic shift of longwave emissions, which evidences their lower polarity. It is one of the characteristic qualities of ketone tautomer emissions occurring in excited states. As follows from literature data, the shortwave emission originates from the enol form, usually stabilised in a polar medium by the formation of hydrogen bonds with surrounding solvent molecules or molecules of other compounds (Scheme 2B, F). After the fluorescence emission, the ground-state ketone tautomer molecule very quickly shifts to the more stable enol form.

At this point, one should also note the very evident contribution of molecular aggregation effects which, in these particular molecules, clearly enhance the observed dual fluorescence effects. As is most apparent in the case of TS, Fig. 1E, a very clear effect of dual fluorescence also emerges in butan-1-ol, i.e. a polar solvent additionally characterised by high viscosity. As such, it facilitates the analysis of aggregation effects visible both in the absorption spectra—as a widening on the longwave side, and emission spectra—evidenced by the considerable decrease in spectral intensity. Hence, the effects of dual fluorescence observed for TSF and TS are in fact related to two distinct factors. The primary factor is ESIPT as the dual emission spectra can also be observed in completely non-polar media where aggregation effects, even if they do occur, are only possible between molecules of the same compound. However, there is also the second significant factor—i.e. aggregation effects that change medium hydrophobicity and significantly enhance the ESIPT effects related to dual fluorescence emission. The contribution of aggregation effects is significant in this case and stems from the effects of AIE. Naturally, as we have verified from the crystallographic perspective in monocrystalline systems of the compounds, we could also be dealing with molecules structured as presented in Scheme 2D, H. After all, such molecules are also capable of aggregation effects given the right medium or simply appropriate concentration. However, in such a case the hydroxyl group would be positioned on the side of the sulphur atom in the thiadiazole ring, which would block proton transfer and prevent ESIPT-induced dual emission.

The quantum yields results (*Φ*_F_) of TB, TS and TSF in the selected solvents confirm that participation in the presented changes in the fluorescence emission spectra is mainly related to ESIPT, but significantly enhanced for selected analogues by processes related to molecular aggregation. As we show in Table S2 in the Supplementary Material (SM), for each of the analogues, the fluorescence quantum yield is the highest in methanol, lower in ethanol, and drastically decreases in butan-1-ol, where for TSF, and above all for TS, the effect of dual fluorescence was observed. The decrease in quantum yield for TSF and TS is related to the ESIPT process, showing the definitely less polar nature of these excited states, which for these analogues, especially TS, is strengthened in the appropriate environment by the process of molecular aggregation.

The effects described above and the related hypotheses are corroborated by the fluorescence excitation spectra presented in Fig. S1 as well as resonance light scattering (RLS) spectra in Fig. S2, both included in the SM, which correspond to the respective emission spectra. The fluorescence excitation (Ex) spectra presented in Fig. S1 were recorded for the same solvents as those discussed in Fig. 1. For the analogues selected in this study, excited state emissions were registered at wavelengths corresponding to short or longwave emission depending on the solvent (the relevant data are provided in Fig. S1). As we know, fluorescence excitation spectra, due to their high selectivity, allow an in-depth insight into specific energy levels that correspond to specific forms of molecules, which are in turn responsible for the given type of emission—short or longwave (Rodembusch et al. 2007). Panel A in Fig. S1 reveals that for TB, only a slight shift of the excitation spectra can be observed, which is in this case related to the type of solvent and the possible presence of minor aggregation effects, as evidenced by changes in spectral intensity. However, the most interesting effects confirming our earlier premise can be observed in Panels B and C. For instance, Panel C reveals (for TS) in the region of ~ 300 nm that the excitation spectra are slightly shifted relative to each other in ethanol and toluene (chloroform), which evidences the existence in the ground state of *cis-* and *trans*-conformers of the enol form, as already observed in the literature (Rodembusch et al. 2007). The visible shift towards longer wavelengths in the *cis-*form relative to the *trans-*conformer is naturally due to the existence of an intramolecular hydrogen bond (Czernel et al. 2020). Moreover, as corroborated by our studies on monocrystalline systems conducted for similar analogues (Hoser et al. 2013, 2018), the *cis-*form is more stable than the *trans-*form as it allows for molecule stabilisation via the intramolecular hydrogen bond (Scheme S1 in the SM). At the same time, the effect of aggregation is particularly clearly visible in the excitation spectra as it enhances the phenomenon of dual emission in TS and TSF. As can be seen in butan-1-ol, the excitation spectra are considerably less intense and their main maximum is shifted to approx. 327 nm. This effect can be observed for both TS and TSF and confirms the contribution of aggregation effects related to AIE as factors responsible for the emergence of dual emission in these molecules. Additionally, it is worth emphasizing that depending on the excitation wavelength there are quite significant differences between the absorption spectra and the corresponding fluorescence excitation spectra. Based on our previous studies, the differences at about 315 nm (for TB) or about 300 nm (for TS and TSF) are related to the presence of *cis*- and *trans*-conformers, and consistent with results reported elsewhere (Bhattacharyya et al. 2020; Heyer et al. 2017; Rodembusch et al. 2007). On the other hand, the longwave differences with a maximum of about 350 nm (for TS and TSF) are associated with the aggregation of these compounds (Czernel et al. 2020; Tong et al. 2015).

The assumption that aggregation related to AIE fluorescence only enhances fluorescence effects induced primarily by the ESIPT effect is further corroborated by the resonance light scattering (RLS) measurements presented in Fig. S2 in the SM. As we can see, RLS spectra are present even for TB where only a single fluorescence emission spectrum is observed regardless of the solvent used. Moreover, in the case of TSF and TS, RLS spectra are observed even in solvents where dual fluorescence does not occur, e.g. ethanol. It should also be underlined that in solvents where the effect of dual fluorescence does take place, a significant increase in RLS spectra is observed—e.g. in butan-1-ol for both TSF and TS. Hence, in the type of molecule, aggregation as such may not be the main factor responsible for the emergence of the discussed effect; however, it is able to significantly improve it under appropriate conditions. In our case, the aggregation enhances the phenomenon of dual fluorescence which is primarily induced by the ESIPT process. It is also noteworthy that the relatively uniform form factor of the RLS spectra may evidence preference for a specific type of molecular aggregate. In accordance with the exciton splitting theory proposed by M. Kasha and based on our analysis of the absorption spectra, we notice most likely the “*card pack*” aggregation, at least in the case of TSF and TS molecules, as mentioned above.

Additionally, the above considerations are supported by fluorescence emission spectra recorded in butan-1-ol at various concentrations of TS and TSF (Figure S3), which clearly show that in the case of shortwave excitation, an increase in compound concentration results in a significant increase in the emission at about ~ 435 nm (in the dual fluorescence spectrum of both TS and TSF). The lower the concentration of the compound the lower the ratio of the intensity of the maxima at 440 and 380 nm. This evidences the impact of aggregation on the fluorescence effect studied.

### Studies of spectroscopic effects in liposomes

In the subsequent part of our study, after the analysis related to the presence of various fluorescence forms in solvent systems, we aimed to determine which one is preferred in membrane model systems for the three selected analogues: TB, TSF, and TS. We also sought to establish how this preference influences the spectroscopic and dynamic properties of the model liposomal membrane depending on the structure of the molecule and its associated aggregation characteristics. To this end, we conducted in-depth studies with the use of electronic absorption spectroscopy, fluorescence spectroscopy, as well as SPR and EIS measurements.

Firstly, the spectroscopic measurements were performed on 1, 3, 5, 10, 15, and 20% mol TB, TSF and TS in a liposomal medium with DPPC. Fig. S4 presents the electronic absorption spectra of TB at the concentration of 10% mol TB in DPPC, relative to changes in the temperature of the liposomal medium. The temperature changes led to slight changes in the shape of the absorption spectra. In this medium, TB is present primarily in its monomeric form, as evidenced by the single, narrow absorption band with the maximum at approx. 290 nm, characteristic of the *π* → π* electronic transition (as already discussed above). This band is only slightly built up at its long wavelength side, indicating that the contribution of aggregation effects in TB is negligible, and related to the **TB** structure. A slight increase in the intensity of the main absorption band is observed as the solution temperature increases and the lipid’ transition from the *L*_β’_ to *L*_α_ phase, after the main phase transition. The long-wave band hardly changes in intensity with a change in the temperature of the main phase transition. Meanwhile, at the main phase transition temperature in lipid, the spectrum slightly shifts towards the short wavelength side, which evidences the fact that as the lipid membrane changes its fluidity, molecules of the compound can more easily engage interactions.

These changes are clearly visible in Fig. S5, which presents the fluorescence emission spectra for 10% mol TB in DPPC, corresponding to the absorption spectra from Fig. S4. We can see that with increasing temperature of the medium, the intensity of the emission spectra is significantly reduced, taking into account the entire range of tested temperatures. For the absorption spectra, a slight enhancement of the band characteristic of aggregated systems was observed. It is that effect that causes the decrease in emission band intensity. However, an enhancing factor may also be the temperature change, which significantly influences the aggregation effects. The above results are corroborated with the fluorescence excitation spectra presented in Fig. S6 and corresponding to the emission spectra in Fig. S5. Here, a relatively significant decrease in spectral intensity can be observed, particularly for temperatures nearing that of the main phase transition, with the effect becoming slightly stronger above the *L*_β_ to *L*_α_ shift temperature. Finally, Fig. S7 presents the RLS spectra corresponding to the results in Figs. S4–S6. These data confirm that, even at the lowest temperatures of the lipid medium, the molecules form both monomeric and aggregated structures, albeit, in the case of this particular analogue, the latter are significantly less common. Above the temperature of the *L*_β′_ to *L*_α_ phase shift, the prevalence of monomeric forms in the lipid is further increased, but aggregated structures remain present to a sufficient extent for the RLS spectra to still show fairly high intensity, roughly 80% of their initial intensity.

**Fig. 2:**
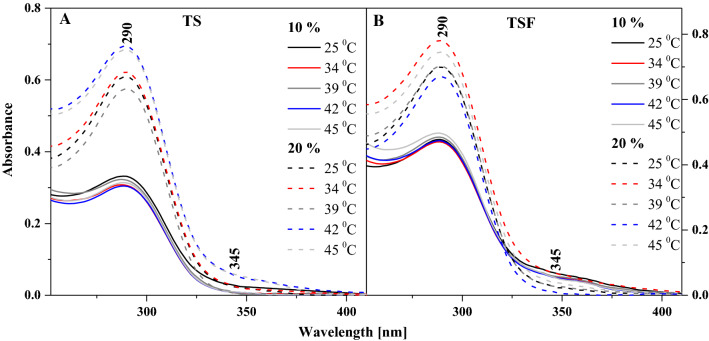
Electronic absorption spectra with changes in temperatures for TS (**A**) and TSF (**B**) in the DPPC medium at the selected mol % concentrations

In the subsequent part of the study, similar measurements were conducted in liposomal systems for the other two analogues, i.e. TSF and TS, whose structures are considerably more interesting. Both TS and TSF have the ability to form a hydrogen bond (Scheme S1), supporting the ESIPT effect. Furthermore, the two compounds show significantly stronger aggregation effects, particularly in the case of TSF whose structure additionally includes a HO_3_S-group. Figure 2 presents selected electronic absorption spectra for TS (Panel A) and TSF (Panel B) at 10% mol of the respective compound in DPPC, at selected temperatures of the liposomal medium. As can be noted, similarly to TB a band with the absorption maximum at approx. 290 nm is present in both these compounds, which is characteristic of the *π* → π* electronic transition in the analysed monomeric form of the molecule. At the same time, the two compounds show a very clear broadening of the bands on the longwave side with a very wide maximum at approx. 350 nm, which evidences the presence of strong aggregation effects. With increasing medium temperature the lipid undergoes a transition from the *L*_β′_ to the *L*_α_ phase, past the main phase transition, where the absorption spectra of TS and TSF show a noticeable increase in the intensity of absorption from the monomeric forms. At the same time, the longwave band noticeably decreases in intensity. Also, the maximum of the absorption spectrum, at temperatures nearing the main phase shift, is shifted in the shortwave direction in both TS and TSF. This is consistent with the observation that the change in the membrane’s fluidity facilitates mutual interactions between the molecules of the analysed thiadiazoles.

**Fig. 3:**
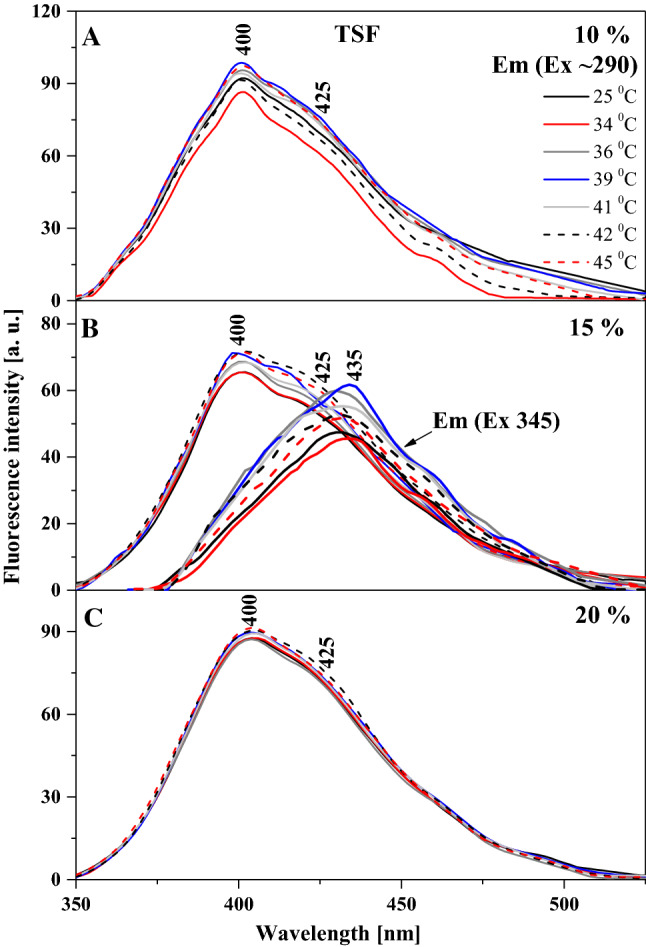
Fluorescence emission spectra for the TSF. Panels A–C show fluorescence emission spectra for different mol % concentration of TSF 10%, 15% and 20%. The emission spectra for analysed samples were obtained at excitation wavelength in the absorption band maximum Em (Ex 290). Additionally, for the concentration of 15% spectra for the excitation wavelength in the absorption band at 345 nm are shown. For clarity of the results, only the spectra obtained at 25, 34, 36, 39, 41, 42 and 45 °C are shown

**Fig. 4:**
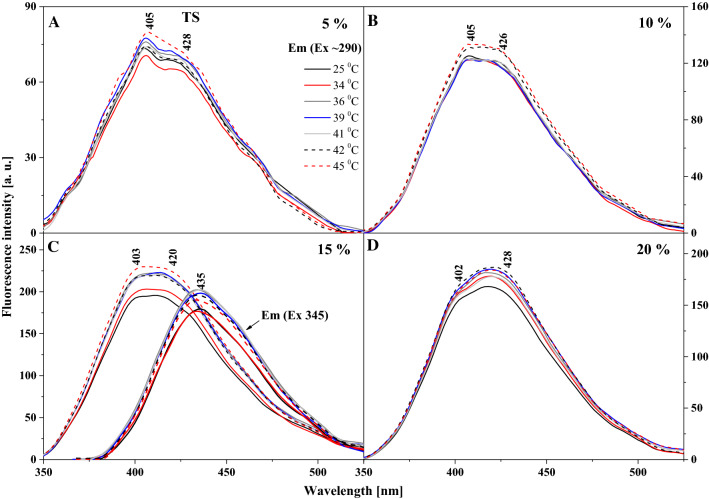
Fluorescence emission spectra for the TS. **A**–**D** show fluorescence emission spectra for different mol % concentration of TS 5%, 10%, 15% and 20%. The emission spectra for analysed samples were obtained at excitation wavelength in the absorption band maximum Em (Ex 290). Additionally, for the concentration of 15% spectra for the excitation wavelength in the absorption band at 345 nm are shown. For clarity of the results, only the spectra obtained at 25, 34, 36, 39, 41, 42 and 45 °C are shown

**Fig. 5:**
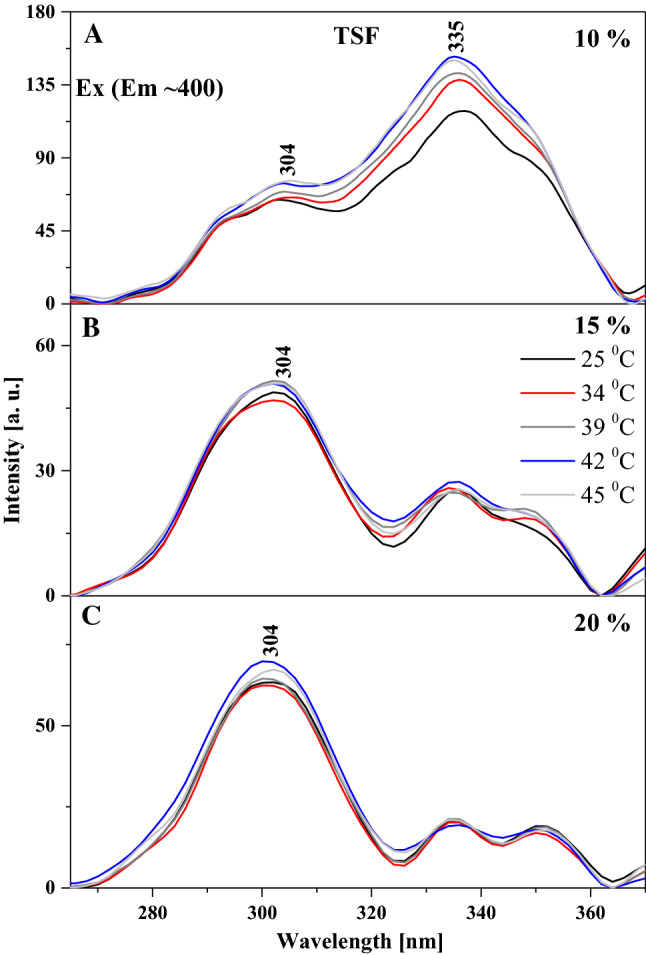
Fluorescence excitation spectra for the TSF. **A**–**C** show fluorescence excitation spectra for different mol % concentration of TSF 10%, 15% and 20%. The excitation spectra for analysed samples were obtained at emission wavelength in the fluorescence band maximum Ex (Em ~ 400). For clarity of the results, only the spectra obtained at 25, 34, 39, 42 and 45 °C are shown

**Fig. 6:**
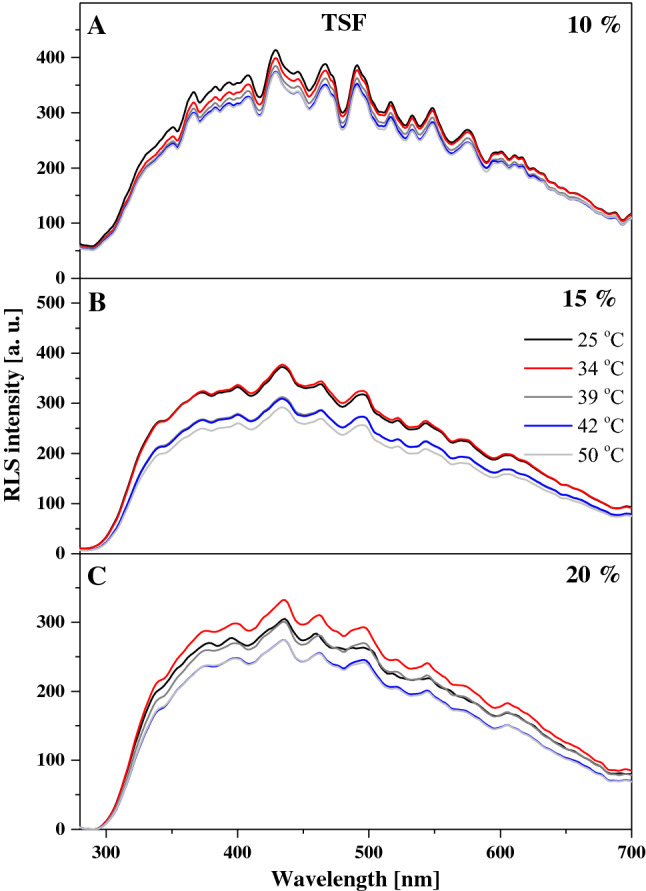
Resonance light scattering (RLS) for the TSF. **A**–**C** show RLS spectra for different mol % concentration of TS 10%, 15% and 20%

Particularly interesting effects are observed in the fluorescence emission spectra for the two analogues, as shown in Figs. 3 and 4. Figure 3 presents the emission spectra corresponding to the relevant absorption spectra in TSF, where we can see that the wide maximum is positioned at approx. 400 nm. In the case of this analogue, with increasing temperature and for the respective tested molar concentrations of the compound in the lipid, we observe a slight upward trend, especially for 15% mol TSF in DPPC (Fig. 3B). The effects are less pronounced but still noticeable for the 10 and 20% molar concentrations of TSF in DPPC. Let us also point out that the absorption spectrum maximum for 10% mol TSF in DPPC is located at approx. 400 nm, i.e. similarly to the parallel spectrum in ethanol where the enol monomeric form of the compound is dominant. However, after the phase shift, the intensity of the band increases at approx. 430 nm due to changes in membrane dynamics, which allow molecules to interact more freely and partially move towards their hydrophobic form, which is more conducive to the ESIPT process. Hence, we observe a relatively noticeable increase in longwave emission with the maximum at approx. 430 nm. The decrease in the intensity of fluorescence emission spectra for 15% mol TSF in DPPC compared to the 10% mol concentration further evidences the impact of aggregation effects. However, a very interesting effect is observed in Fig. 3B, where for the excitation at 345 nm we observe a shift of the emission band from 400 to 435 nm, similar to what we previously described for TSF. Clearly, we can see the emission band also related to the ESIPT process, not only to the above mentioned increase in the emission on the longwave side for the excitation at 290 nm. Depending on the excitation, we observe emission bands related to the emission from the excited enol and keto form. However, in the liposomal environment a shifted emission band is observed in the longwave excitation from the absorption spectra, which proves quite a significant influence of aggregation effects on this effect, similar to that for the TSF analogue. The same is also corroborated by the fluorescence excitation spectra presented for TSF in Fig. 5, as well as the RLS spectra shown in Fig. 6. In the case of excitation spectra for TSF in the liposomal system, we see bands characteristic of both aggregated systems—on the longwave side—and monomeric forms—on the shortwave side. In this case, after the lipid phase shift, we observe an increase in the band intensity, particularly for lower concentrations of the compound in the lipid. At concentrations higher than 15 or 20% mol TSF in DPPC, the trend is also present, but the aggregated forms, due to their number, maintain the high intensity of their bands. RLS spectra lose their intensity with increasing sample temperature, which is confirmed by the fact that the number of various aggregated forms decreases and the number of monomeric forms increases. Nonetheless, the RLS spectra continue to appear, which simultaneously evidences the presence of aggregated forms and their impact on the analysed effects.

**Fig. 7:**
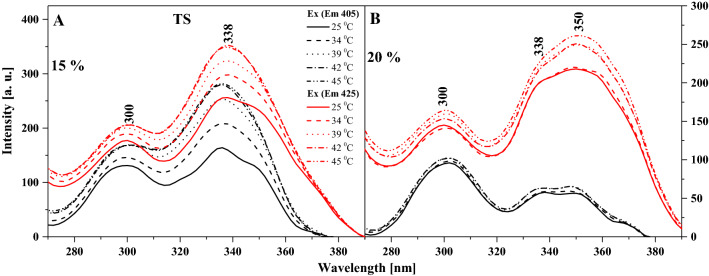
Fluorescence excitation spectra for the TS. **A**-B show fluorescence excitation spectra for different mol % concentration of TS 15% and 20%. The excitation spectra for analysed samples were obtained at emission wavelength in the fluorescence band maximum Ex (Em 405) and Em (Ex 425). For clarity of the results, only the spectra obtained at 25, 34, 39, 42 and 45 °C are shown

**Fig. 8:**
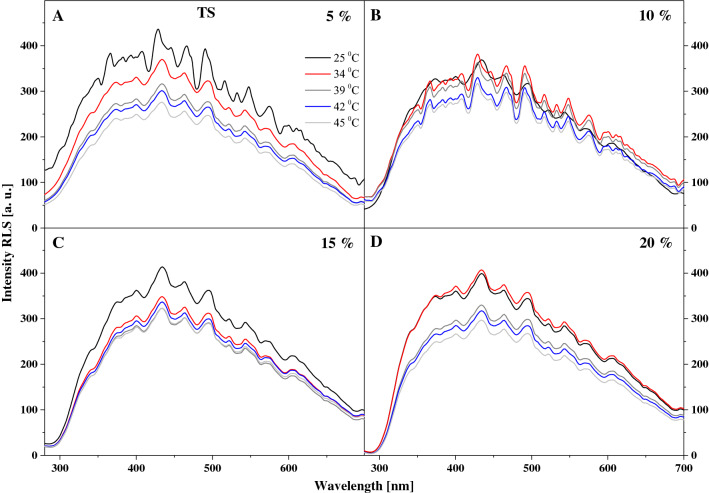
Resonance light scattering (RLS) for the TS. **A**–**D** show RLS spectra for different mol % concentration of TS 5%, 10%, 15% and 20%

To conclude the discussion on the observed spectroscopic effects in the liposomal system, Fig. 4 presents fluorescence emission spectra, corresponding to the absorption spectra in Fig. 2A, for various molar concentrations of TS in DPPC. In this case, the observed changes are very similar to those reported for TSF but more accentuated. Firstly, as the temperature of the lipid medium increases, we can see an increase in the intensity of emission bands, particularly above the temperature of the main phase transition of DPPC related to the *L*_β′_ to *L*_α_ transition. Similar to TSF, also in this case, we observe clearly defined maxima in the emission spectra, one at approx. 400 nm and the other at approx. 428 nm. The bands with the maximum at approx. 400 nm are characteristic of the enol forms of TS molecules, usually dominant in low concentrations of the compound and at temperatures below the main phase transition of the lipid. However, for higher concentrations of TS and with the temperature increasing towards the phase shift, the band with the maximum at approx. 400 nm visibly loses intensity (Fig. 4B, C), while the band with the maximum at 428 nm becomes dominant (Fig. 4D) for short wavelength excitation from absorption spectra at 290 nm. The changes clearly evidence a correlation between the monomer/dimer (or aggregate) equilibrium and the changes in lipid membrane fluidity. Figure 4D clearly shows that for extremely high molar concentrations of the compound in the lipid, the dominant fluorescence band becomes the one attributed to the ketone tautomeric form of TS in solvent experiments. It is worth emphasizing that the analogous effect for the TSF is also presented for TS in Fig. 4C, which also shows the emission spectra with excitation at 345 nm as mentioned above. This excitation corresponds to the characteristic bands of the aggregated forms of TS in a given environment. However, the emission band corresponds to the emission from the TS ketone tautomer and the presence of the ESIPT process in the liposomal environment. In Panel C we observe separate emission of the enol tautomer with a maximum at about 403 nm that shifts, even with this shortwave excitation towards the longwave side. As well as emissions from the excited ketone tautomer with a maximum around 435 nm, this is also clearly related to the impact of aggregation effects on this process. It should be noted, however, that the effects are enhanced by aggregation, as evidenced in Figs. 7 and 8, which present the excitation and RLS spectra for TS in the liposomal system, and as previously discussed in detail with relation to Fig. 1. The fluorescence excitation spectra show that as the excitation wavelength increases, the intensity of fluorescence increases on the long wavelength side of the spectra, which represent the bands associated with the aggregated forms of the compound. Meanwhile, RLS spectra decrease in intensity upon temperature increase, which points at a decreased number of various aggregated forms. Nonetheless, the RLS spectra continue to appear throughout the experiment, which corroborates the still significant contribution of aggregated forms to the described fluorescence effects.

It is noteworthy that Fig. S8 presents the relationships emphasizing the influence of selected compounds on changes in the fluidity of the liposomal membrane, and which in the next part of this work are described in more detail using surface plasmon resonance and electrochemical impedance spectroscopy studies. As we can see in Fig. S8, where the relationship between the positions of the absorption maximum as function of temperature for selected analogues is presented, we observe that TB behaves quite opposite to TS and TSF within the range of the main phase transition temperature, which will be reflected later in the work through SPR and electrochemical impedance spectroscopy studies. Thus, TSF and TS compounds have a much greater impact on the liposomal membrane. To sum up briefly, the above results show that organizational changes related to the effects of ESIPT, are present especially for TS and TSF, which we can observe even in the lipid environment. These changes are enhanced by the aggregation process, which in an environment with such variable hydrophobicity may enhance the observed fluorescent effects around the main lipid phase transition, and are associated with an increase in diffusional freedom inside the hydrophobic lipid layer. The position of the thiadiazole molecules in the liposomal membrane, especially TS and TSF, significantly increases the energy barrier to the rotation and gauche-trans isomerization of neighbouring lipid molecules. Such interactions are related to the delicate stiffening of the lipid membrane, which is related to the shift of the main phase transition towards higher temperatures, which is also influenced by the concentration of the compound in use.

In addition, Fig. S9 shows selected FTIR spectra for TS and TB in the lipid system at temperatures below and above the main phase transition. The details of the vibration assignment of the pure DPPC and DPPC with TB and TS compounds are presented in Table S4. As can be seen, selected thiadiazoles mainly affect changes in the bands characteristic of polar lipid heads, such as: stretching vibrations of the PO^−2^ groups (~ 1250 cm^−1^), C–O–P–O–C (~ 1090 cm^−1^), or N^+^(CH_3_)_3_ (968 cm^−1^). The compounds, mainly TS, only slightly influence changes in the hydrophobic region associated with the vibrations of –CH_2_ and –CH_3_ groups (slight change at 1467 or 1378 cm^−1^). It can be seen that the choline band centered around 970 cm^−1^ shifts toward higher frequencies for TB and TS compared to pure DPPC lipid (see figure S10 in supplementary materials). It indicates that there are interactions between the TB and TS molecules and the hydrophilic regions of the lipids. For TS a noticeable increase of frequencies at higher concentrations (15%) is observed. The same effect is observed on the position of the 1068 cm^−1^ (v C–O–PO_2_^−^) which is presented in Fig. S10 panel B. The positions of CH_2_ stretching vibrations (2850, 2950 cm^−1^) for TB and TS remain almost unchanged for all the concentrations measured (Fig. S10 panels C and D). Changes in the area of the groups associated with the hydrophobic part of the liposomal structure are enhanced for TS mainly at temperatures above the main lipid phase transition.

Knowing from fluorescence studies that at a concentration of 10% mol, intramolecular interactions are noticeable, this concentration was used for all analogues in a liposomal medium with DPPC, for both SPR and EIS measurements.

### Surface plasmon resonance study

Surface plasmon resonance (SPR) studies enable the label-free characterization of biomolecular interaction processes, since they lead to small changes in the refractive index in the vicinity of a metal surface, such as gold (Green et al. 2000). Surface plasmons are electromagnetic waves, generated due to the differences between the dielectric constant (ε) of each medium at the water/metal interface. A beam of light will hit the metal surface at a specific incident angle, the SPR angle, which in the presence of biomolecules will change, due to changes in the refractive index (Abbas et al. 2011).

SPR was used to characterize DPPC phospholipid membranes and their behaviour in the absence and presence of three 1,3,4-thiadiazole analogues as a function of temperature. The lipids being an enclosed environment and in the vicinity of a hydrophobic gold surface, the DPPC vesicles should arrange themselves into a so-called vesicle film.

**Fig. 9:**
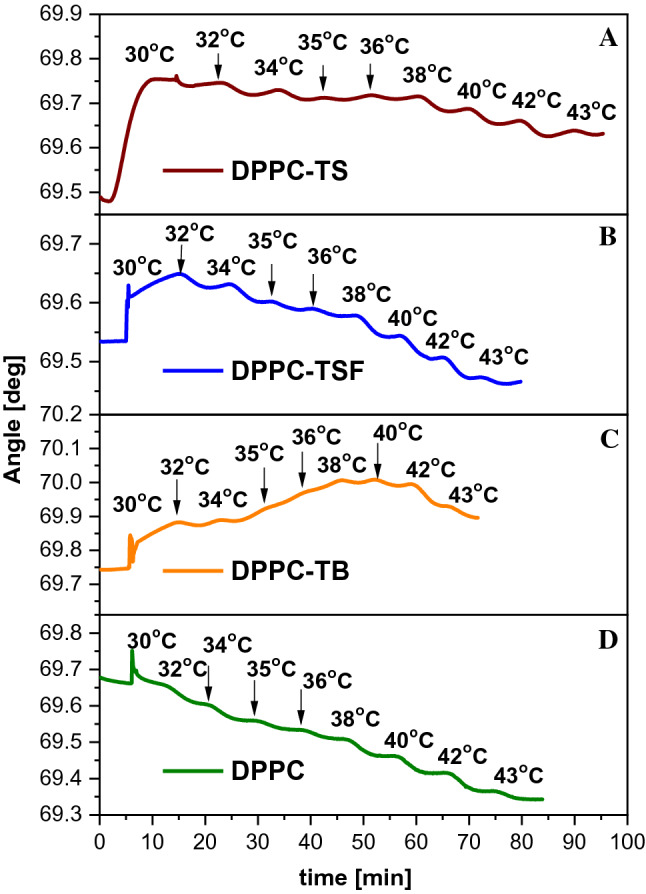
Sensograms monitoring the angle shift as function of temperature in time for **A** DPPC-TS, **B** DPPC-TSF, **C** DPPC-TB and **D** DPPC

For quantitative probing of the DPPC-thiadiazole systems, the changes in SPR angle were monitored. These variations depend on the changes of the dielectric properties of the DPPC vesicle-film formed in the vicinity of the gold surface. The DPPC and DPPC-compound systems were added into the preheated (30 °C) electrochemical SPR-cell after approx. 5 min, upon PBS buffer signal stability. The flow was stopped, and after achieving stability upon lipid addition, the temperature was stepwise increased, as shown in Fig. 9. The PBS medium may form a thin layer of solvent between the gold surface and the vesicle arrangement. Thus, the thickness and optical properties of the vesicle-film will change as function of temperature, causing an SPR angle shift. From theory, SPR angle shifts towards higher degrees suggests mass adsorption (chemical or electrostatic binding to the gold surface), while a shift towards lower degrees highlights the changes of the vesicle film itself (DPPC-compound system).

In all our study scenarios, the SPR angle shifts towards lower degrees with increasing temperature, except the DPPC-TB system. The decrease of the SPR angle shift is proportional to the decrease of refractive index of DPPC vesicle-film (*n*), consequently with its ε. In this situation the optical thickness of the vesicle layer keeps decreasing with increasing temperature, suggesting an increase in the membrane fluidity.

We will comment on each system separately, starting with the DPPC-TSF system (Fig. 9B). Here, the changes occur in two steps: first, there is an SPR angle increase for temperature variation from 30 to 32 °C, followed secondly by a slow decrease between 34 and 40 °C, and a more pronounced decrease after the main transition temperature was reached. With increasing fluidity of the membrane, TSF molecules should interact more easily with the membrane. We noticed though, that the SPR angle is lower for the DPPC system in comparison to the DPPC-compound systems. This means that the DPPC membrane fluidity is more pronounced in the absence of the three analogues. FTIR studies showed that the analogues interact more strongly with the hydrophilic part of the DPPC system (polar lipid heads), rather than the hydrophobic region, increasing membrane fluidity. Even though the DPPC-compound systems form a more rigid film, as shown by SPR, they all present a decrease of the SPR angle starting at a temperature of 38 °C, suggesting the interaction of the compounds with the membrane. It is also possible that the membranes rupture; and since we know that the compounds preferably interact with the polar lipid heads, they have “a free” sulphur atom, which may chemically adsorb to the gold layer. The high affinity of thiolates towards gold is well known in the literature; thus, if the compounds penetrate the membrane, they will form a chemical bond on the gold surface.

The behaviour of the DPPC-TSF complex shows the steepest SPR angle decrease for temperatures between 38 and 43 °C (Δ_TSFshift_ = 0.1033 deg); where the DPPC system has a shift of 0.1461 degrees for the same temperature range. TS behaves similarly, where the decrease in angle shift is continuous, with a plateau-like behaviour for temperatures between 35 and 38 °C. The same decrease will be seen for the electrical capacitance of the membrane—CPE_m_ in subsequent EIS measurements. The SPR angle shifts towards lower degrees, but in smaller intervals (Δ_TSshift_ = 0.0769) for temperatures between 38 and 43 °C. Herewith, the vesicle-film optical thickness changes less, proportional to the decrease of *ε*. TS lacks the HO_3_S-group present in TSF, which may lead to a lower degree of intramolecular interaction. This is also in accordance with FTIR measurements, which show that TS is more prone to interact with the hydrophobic region of the membrane. For the DPPC-TB system, a different behaviour can be noticed. For temperatures between 30 and 40 °C, there is an increase of the angle shift with a plateau between 32 and 34 °C. Starting with 40 °C, a turning point occurs, and the SPR angle starts shifting towards lower values (Δ_TBshift_ = 0.0784 degrees for temperatures between 40 and 43 °C). This phenomenon can be well observed in Panel C of Fig. 9. There is a slight decrease from 40 to 42 °C, followed by a steep decrease at 43 °C. When compared to EIS data, here, the DPPC-TB system has also a different behaviour in terms of electrical resistance of the membrane, which will be better highlighted in the next section. Thus, the fluidity of the membrane does not significantly influence the intramolecular interactions, highlighting the fact that TB was shown previously not to form hydrogen bonds as TS and TSF do.

### Electrochemical impedance spectroscopy study

Due to the placement of the electrochemical-SPR cell, the relative ordered arrangement of the vesicles into a film near to the gold surface will be viewed as a membrane and denoted as such in this section. EIS was employed in order to determine the effect of the 1,3,4-thiadiazole compounds on the membrane conductivity as function of temperature. The characterization of the phenomena at the SPR chip-electrolyte interface adds information about electron transfer and diffusional processes highlighted by the changes which occur in the values of the electrical equivalent circuit components. In these series of measurements, we refer to electrolyte as the sample containing the DPPC liposomal system in 10 mM PBS, as such, and enclosing a concentration of 10% mol of TS, TSF or TB. Two aspects can be monitored using this technique: the influence of temperature on the membrane and the interaction of compounds with the membrane.

**Fig. 10:**
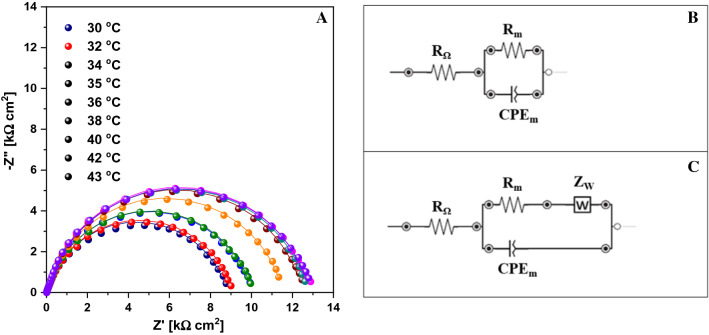
**A** Nyquist plot in 10 mM PBS, pH 7.4, applied potential of -0.35 V vs*.* Ag for DPPC-TSF complex as function of increasing temperature values from 30 to 43 °C (lines represent the fittings). **B** Corresponding electrical equivalent circuit. **C** Electrical equivalent circuit for DPPC system (Nyquist plot not shown)

**Fig. 11:**
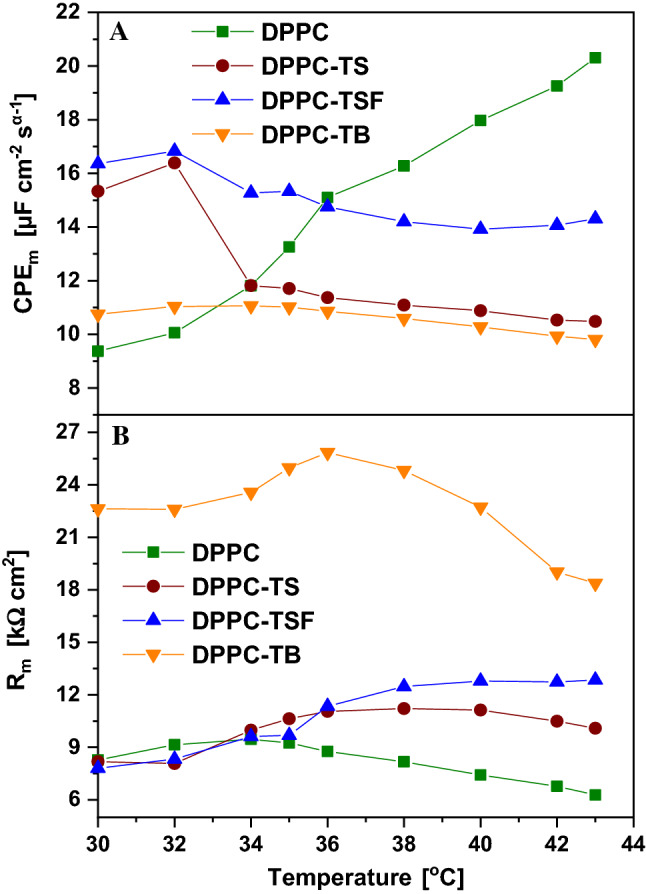
Variation of conductance and capacitance as function of temperature of the four samples: DPPC, DPPC-TS, DPPC-TSF and DPPC-TB for: **A** membrane capacitance (CPE_m_) and **B** membrane resistance (*R*_m_)

Figure 10A represents the complex plane plot (Nyquist plot) for the DPPC-TSF sample, as a function of temperature. The electrical impedance of the membrane is usually determined by the dielectric properties of the phospholipid membrane (Valincius et al. 2012). For a better understanding of the membrane resistance and capacitance, all impedance data were fitted to an electrical equivalent circuit, depicted in Fig. 11, Panels B-C. The electrical capacitance of the membrane is non-ideal, therefore being modelled as a constant phase element (CPE) with impedance, following the expression: *Z*_CPEm_ = [(*Ciω*)^αm^]^−1^, where *C* is the ideal capacitance, *ω* the radial frequency and the exponent αm reflects the surface uniformity (vesicle film), which can vary between 1.0 for a perfect smooth surface and 0.5 for a non-uniform, rough surface (David et al. 2018). To highlight the influence of the three analogues on the DPPC liposomal system, it was necessary to record separately the spectra of the DPPC liposomes. These spectra were fitted with an electrical circuit consisting of a cell resistance *R*_Ω_ (electrical resistance of the electrolyte and all connectors), in series with a parallel combination of the membrane resistance *R*_m_ (responsible for the charge transfer through the membrane) further in series with a Warburg element (*Z*_w_), and the membrane capacitance (CPE_m_) (Fig. 10C). When the DPPC liposomes enclosed any of the three 1,3,4-thiadiazole compounds, a simplified circuit could be used to fit the spectra, consisting of a cell resistance *R*_Ω_, in series with a parallel combination of the membrane resistance *R*_m_ and CPE_m_ (Fig. 10B). This simple circuit could be used due to very simple impedance diagrams, having the form of impedance semicircles. Such a circuit is characteristic for an artificial lipid membrane without any defects (pores, ionophore channels) (Naumowicz and Figaszewski 2005).

The Warburg impedance becomes important in the low frequency region (below 10 Hz), where diffusing reactants such as ions, need to diffuse farther through the electrolyte, highlighting diffusion-limited processes at the electrode surface. This is a well-known behaviour for gold surfaces, where a semi-infinite diffusion is assumed (Chilom et al. 2020). In the presence of the DPPC liposomes, the influence of the Warburg impedance is still present, indicating however lower values compared to gold (Table S3).The *Z*_W_ values keep decreasing with temperature (from 1.34 to 0.79 kΩ cm^2^), indicating the depletion of the diffusion layer once membrane fluidity is reached. Thus, we assume that the liposomes nicely organize themselves in an ordered arrangement in the close vicinity of the gold chip surface, as the membrane vesicle-film mentioned before. An important aspect to be considered here is also the fact that the gold surface is hydrophobic, which constrains the ordered arrangement of the hydrophobic vesicles into a film, shaped after the morphology of the gold surface. When TS, TSF or TB are enclosed in the DPPC liposomes, the effect of the impedance is determined by different physical phenomena (conductance and capacitance). These are rather highlighted by the RCPE parallel combination of circuit elements, changing the behaviour of the membrane (change in hydrophobicity which hinders the diffusional processes and increases charge transfer resistance). The conductance of the membrane vesicle-film is characterized by a low charge transfer resistance, while its capacitance depends on charge accumulation. For all 1,3,4-thiadiazole compounds, we have a less conductive membrane, as highlighted by the equivalent circuit element values in Table S3, having significantly increased *R*_m_ values and overall decreased CPE_m_ and *α*_m_ values, as compared to the DPPC liposomes.

The changes recorded for each circuit element as a function of temperature, allow us to monitor the changes of the membrane properties with increasing temperature for each system. Since the membrane conductance, (*R*_m_) and capacitance are of greatest importance, Fig. 11A, B shows the variation of these parameters as a function of temperature (according to the values in the table). For the DPPC vesicle-film, the charge transfer through the vesicles slightly increases, reaching a maximum of 9.46 kΩ cm^2^ at 34 °C. This means that, near the pre-transition temperature, the DPPC vesicle-film has the lowest conductivity. As temperature increases, the membrane conductance slowly and continuously decreases, indicating that the membrane slightly gains fluidity with increasing temperature. The capacitance significantly increases with increasing temperature, and together with the increase of the *α*_m_ coefficient, we can conclude that the vesicles, due to increased fluidity, form a uniform layer changing its dielectric properties.

The presence of the 1,3,4-thiadiazole compounds highlights some critical temperatures (in the vicinity of the pre-transition and main transition temperatures of DPPC) where significant changes in the electrical circuit element values occur. In the case of *R*_m_, a similar behaviour of the DPPC-TS and -TSF complexes can be noticed. With increasing temperatures, the resistance values slowly increase up to 40 °C (close to the main transition temperature of 41 °C), after which for TSF they remain constant (~ 12 kΩ cm^2^), whereas for TS they slightly decrease. Compared to DPPC, the resistance values are higher, meaning a weaker electron transfer rate in the presence of TS and TSF, and herewith a less conductive membrane. In the presence of TB, the resistance values are the highest, more than double up to 36 °C (25.84 kΩ cm^2^), but with a very high decrease rate after the pre-transition temperature, decreasing up to 18.37 kΩ cm^2^ at 43 °C. When compared to the SPR angle decreased trend of the DPPC-TB system, the same similarity arises (a slow, almost constant increase rate between 36 and 40 °C, followed by a steep decrease rate after 40 °C. Thus, membrane fluidity increases while membrane resistance decreases, allowing a better electron transfer, hence not good enough compared to the other two compounds. Thus, TB molecules hinder most the charge transfer through the membrane, a fact which was attributed to the presence of a single functional group (–NH_2_) in its structure; where TS has an additional hydroxyl group (–OH) and TSF has both –OH and –SO_3_H.

In terms of capacitance, the behaviour is different for each of the three compounds. For TS we have a significant decrease from 32 to 34 °C, after which CPE_m_ slowly decreases, while α_m_ increases, suggesting the formation of a more uniform vesicle film, as membrane fluidity increases with temperature. A slightly different behaviour of the capacitance impedance can be observed for TSF, where a decrease occurs from 30 to 38 °C (from 16.36 to 14.20 µF cm^−2^ s^α−1^), and then remains constant for higher temperatures. The surface uniformity is the lowest for the DPPC-TSF system, varying between 0.855 and 0.865, suggesting the formation of a thicker, inhomogeneous film, with a higher resistivity than TS. For the last compound, TB, CPE_m_ values are constant in the 30–34 °C temperature range, starting to decrease slowly in the range 36–43 °C. The changes in *α*_m_ values are also very low, suggesting that the film thickness and uniformity does not change much with temperature changes. Given the high values of the resistance of the DPPC-TB complex, TB molecules hinder most the charge transfer through the membrane, being least suitable for interacting with the DPPC liposomal system. To better highlight the differences at the transition temperatures of 36 and 42 °C for the DPPC liposomal system in the absence and presence of the three 1,3,4-thiadiazole compounds, the corresponding Nyquist spectra is shown in Fig. S11A, B. In addition to the Nyquist plots, Bode plots (impedance and phase) are shown in Fig. S11 C-F and they allow us to better understand the processes associated with the DPPC vesicle-film formed near the gold surface. The impedance response as a function of frequency (panels C and E) provide information on the kinetic phenomena. Usually, at high frequencies, electron transfer kinetics can be monitored, while at intermediate and low frequencies, mass transfer and diffusion processes are highlighted. For all Bode plots, we can see that in the high frequency region the impedance is dominated by a resistive behaviour, where at very high frequencies the phase is 0°. With increasing frequency, the influence of CPE_m_ on the impedance becomes important, and the phase starts to shift, increasing to a maximum of ~ 70° for both temperatures. In the low frequency region, the impedance decreases with frequency, with the most significant decrease for TB. This decrease is attributed to the lack of ion penetration through the membrane. On the phase plots, we can see that the effect of capacitance (CPE_m_) decreases, the phase shifting again towards 0°. The broad shape of the phase angle is attributed to the presence of overlapping time constants of the processes at the DPPC vesicle-film formed near the gold surface. For DPPC, we have a plateau in the intermediate frequency region, but upon adding the 1,3,4-thiadiazole compounds, shifting peaks can be observed and were attributed to diffusion processes through the vesicle-film. These diffusion processes are characteristic for each of the three analogues, and could be attributed to the intramolecular interactions and aggregation processes, highlighted by the spectroscopic techniques. Of great importance in this study, is the fact that the variation of the CPE impedance can be correlated with the variation of the dielectric permittivity (*ε*) of the vesicle-film formed at the gold surface. It is known that the capacitance depends on the dielectric permittivity and thickness of the dielectric sheet of the bilayer. Thus, we can correlate the decrease of capacitance values with the decrease of the vesicle-film thickness and the decrease of the dielectric permittivity of the membrane vesicle-film. These results are also in accordance with the findings using SPR, where the SPR angle shift towards lower or higher angles suggests the variation of the vesicle-film thickness, due to membrane fluidity or liposome accumulation at the gold surface, changes in the equilibrium between monomer and aggregated forms or the lipids’ transition phases.

## Conclusions

The presented results of the conducted measurements of absorption and electronic fluorescence spectra indicate the emergence, in the emission spectra of the TS and TSF analogues, of the effect of dual fluorescence, mainly in non-polar solvent media. In non-polar media, the phenomenon is induced by the ESIPT process, typically occurring in these and other structurally similar molecules. This dual fluorescence effect can also be observed for the mentioned analogues in the context of polar solvents, however, only when the processes of molecular aggregation additionally enhance the phenomenon induced by excited-state proton transfer, in this case associated with the process AIE fluorescence. The structure of the two analogues is clearly conducive to ESIPT processes, which, under the right conditions, coincide with aggregation effects related to AIE. For the TB analogue used in the study, the effects of dual fluorescence were not observed, given its inability to form a hydrogen bond due to the absence of a hydroxyl group in the analogue’s resorcinyl moiety.

The selected analogues were also used in spectroscopic studies conducted in liposomal systems with DPPC. Depending on the substituent and the associated conductivity to ESIPT or aggregation, the compounds were present in a number of forms in the liposomal system. The predominance of forms such as monomers or aggregated systems was also significantly dependent on the phase state of the liposomal membrane as well as the given compound’s concentration. The selected analogues differing considerably in terms of their spectroscopic properties had a very strong influence on the dynamic of the liposomal membrane, particularly at temperatures related to the main phase transition (*L*_β′_ to L_α_) in the lipid.

Further SPR and EIS studies conducted as well in the DPPC liposomal system focus on highlighting the interaction between the liposomal system and the three 1,3,4-thiadiazole analogues. The SPR measurement results could be easily correlated with EIS data, due to changes in dielectric properties of the membrane vesicle-film. Both techniques underline the changes in membrane fluidity with increasing temperatures, and in both cases, TB molecules hinder most the charge transfer through the liposomal membrane, showing the lowest interaction. Both TS and TSF exhibit a similar behaviour, enabling a better electron transfer through the membrane, TS enabling a slightly better conductivity through the membrane than TSF, especially in the fluid-phase.

It is noteworthy that this comprehensive study conducted with the use of selected molecular spectroscopy methods, further complemented by surface plasmon resonance and electrochemical impedance spectroscopy, confirmed the high potential of the discussed group of compounds for applications in molecular biology. They may be utilised as highly sought-after fluorescence probes whose properties can be freely adapted depending on the structure of the substituent system, e.g. to limit their aggregation capacity. Fluorophores undergoing rapid excited-state physicochemical changes can be used for bioimaging due to their high sensitivity and fast response.

## Supplementary Information

Below is the link to the electronic supplementary material.

Supplementary file1 (DOCX 2187 KB)
