# Supplementary material for: Insight into dual fluorescence effects induced by molecular aggregation occurring in membrane model systems containing 1,3,4-thiadiazole derivatives
Source: Eur Biophys J. 2021 Sep 13;50(8):1083–101. doi: 10.1007/s00249-021-01569-7 (PMC8566415; doi:10.1007/s00249-021-01569-7)
Supplement: Supplementary file 1 — Supplementary file1 (DOCX 2187 KB) [file 249_2021_1569_MOESM1_ESM.docx]

**Insight into dual fluorescence effects induced by molecular aggregation occurring in membrane model systems containing 1,3,4-thiadiazole derivatives**

Melinda David^a^, Iwona Budziak-Wieczorek^b^, Dariusz Karcz^c^, Monica Florescu^a^*, Arkadiusz Matwijczuk^d^*

^a^Faculty of Medicine,Transilvania University of Brașov, Brașov 500019, Romania

^b^Department of Chemistry, University of Life Sciences in Lublin, 20-950 Lublin, Poland

^c^ Department of Chemical Technology and Environmental Analytics (C1), Faculty of Chemical Engineering and Technology, Cracow University of Technology, Warszawska 24, 31-155 Kraków, Poland ^d^Department of Biophysics, University of Life Sciences in Lublin, Lublin, Poland

**Correspondence**: arkadiusz.matwijczuk@up.lublin.pl (A.M.); Tel.: +48-814-456-937 (A.M.); Fax: +48-814-456-684 (A.M.); florescum@unitbv.ro (M.F.); Tel.: +40-742-663-420 (M.F.)

| **A** | **B** |
| --- | --- |
|  |  |

**Scheme S1.** Chemical structure of compounds: sulfosalicylic thiadiazol (TSF) and salicylic thiadiazol (TS) with H-bond.

**Figure S1.** Fluorescence excitation spectra of TB (panel A), TSF (panel B) and TS (panel C) dissolved in ethanol, butan-1-ol, toluene and chloroform. The excitation emission was recorded at wavelengths corresponding to the emission maximum. The spectra were measured at room temperature.

**Figure S2.** Resonance light scattering spectra (RLS) of TB (panel A), TSF (panel B) and TS (panel C) dissolved in ethanol, butan-1-ol, toluene and chloroform. The spectra were measured at room temperature.

**Figure S3.** Fluorescence emission spectra of TS and TSF dissolved in butan-1-ol. For TS excitation at 313 nm (panel A), excitation at 335 nm (panel B); for TSF excitation at 317 nm (panel C), excitation at 335 nm (panel D). Measurements were carried out for six different dilution sample.

**Figure S4.** Electronic absorption spectra with changes in temperatures for TB in the DPPC medium at the 10 mol % concentrations.

**Figure S5.** Fluorescence emission spectra for the TB at a 10 mol % concentration relative to DPPC lipid. The emission spectra for analysed samples were obtained at excitation wavelength in the absorption band maximum Em (Ex 290). In order to clarity of the results, only the spectra obtained at 25, 34, 36, 39, 41, 42 and 45ºC are shown.

**Figure S6.** Fluorescence excitation spectra for the TB at a 10 mol % concentration relative to DPPC lipid. The excitation spectra for analysed samples were obtained at emission wavelength in the fluorescence band maximum Ex (Em 382). In order to clarity of the results, only the spectra obtained at 25, 34, 39, 42 and 45ºC are shown.

**Figure S7.** Resonance light scattering (RLS) for the TB at a 10 mol % concentration relative to DPPC lipid.

**Fig. S8.** Relationship between the position of the absorption maximum in dependence of temperature for TB (10 % mol), TS (20 % mol) and TSF (20 % mol).

**Fig. S9.** The ATR-FTIR spectra of DPPC lipid, TB and TS in the form of the dry film for 5 and 15 mol% in DPPC at room temperature 25°C (panel A) and after heating to 45 °C (panel B). All spectra were normalized to the intensity of the band at 2918 cm^-1^ corresponding to the C-H_2_ stretching vibration bands.

**Fig. S10.** Temperature dependence of the position of the main absorption maximum for TS and TB at different molar concentrations in the DPPC liposomes (panels A-D). Red circles indicate the higher temperature 45°C and black squares indicate the lower temperature 25°C.

**Fig. S11.** Impedance spectra recorded in 10 mM PBS, pH 7.4, applied potential of -0.35 V *vs*. Ag for all samples at temperature values of 36°C and 42°C (solid lines represent the fittings). A-B: Nyquist plots, C-D: Bode impedance plots and E-F: Bode phase plots.

**Table S1.** Physical constants of solvents. Dielectric constant ε and index of refraction n of the solvents.

| Compound | Solvent | Max Abs [nm] | Max Abs [cm^-1^] | Max Em [nm] | Max Em [cm^-1^] | SS  [nm] | SS  [cm^-1^] | e | n |
| --- | --- | --- | --- | --- | --- | --- | --- | --- | --- |
| **TB** | Ethanol | 302 | 33112.583 | 379 | 26385.224 | 77 | 6727.359 | 25.3 | 1.3594 |
|  | Butan-1-ol | 302 | 33112.583 | 377 | 26525.199 | 75 | 6587.384 | 17.8 | 1.3993 |
|  | Toluene | 308 | 32467.532 | 380 | 26315.789 | 72 | 6151.743 | 2.38 | 1.4969 |
|  | Chloroform | 295 | 33898.305 | 373 | 26809.651 | 78 | 7088.654 | 4.81 | 1.4429 |
| **TS** | Ethanol | 315 | 31746.031 | 395 | 25316.455 | 80 | 6429.576 |  |  |
|  | Butan-1-ol | 316 | 31645.570 | 406 | 24630.542 | 90 | 7015.028 |  |  |
|  |  |  |  | 440 | 22727.273 | 124 | 8918.297 |  |  |
|  | Toluene | 321 | 31152.648 | 410 | 24390.244 | 89 | 6762.404 |  |  |
|  |  |  |  | 430 | 23255.814 | 109 | 7896.834 |  |  |
|  | Chloroform | 282 | 35460.992 | 408 | 24509.804 | 126 | 10951.190 |  |  |
| **TSF** | Ethanol | 316 | 31645.570 | 400 | 25000.000 | 84 | 6645.570 |  |  |
|  | Butan-1-ol | 318 | 31446.540 | 412 | 24271.845 | 94 | 7174.696 |  |  |
|  |  |  |  | 435 | 22988.506 | 117 | 8458.035 |  |  |
|  | Toluene | 322 | 31055.901 | 406 | 24630.542 | 84 | 6425.359 |  |  |
|  |  |  |  | 435 | 22988.506 | 113 | 8067.395 |  |  |
|  | Chloroform | 318 | 31446.541 | 407 | 24570.024 | 89 | 6876.516 |  |  |

**Table S2.** The plane under the emission curve, the value of absorbance at the excitation wavelength and fluorescence quantum yield for TB, TS and TSF in methanol, ethanol and butan-1-ol.

|  |  | **TB** |  |  | **TS** |  |  | **TSF** |  |
| --- | --- | --- | --- | --- | --- | --- | --- | --- | --- |
| Solvent | I^a^ | λ | $\Phi_{F}$ | I^a^ | λ | $\Phi_{F}$ | I^a^ | λ | $\Phi_{F}$ |
| Methanol | 1639.745 | 280 | 0.0231 | 8631.015 | 292 | 0,1164 | 4532.147 | 295 | 0.0603 |
| Ethanol | 1646.977 | 282 | 0.0241 | 3146.632 | 293 | 0,0443 | 2273.454 | 293 | 0.0321 |
| Butan-1-ol | 1025.447 | 278 | 0.0161 | 578.828 | 296 | 0,0085 | 590.369 | 296 | 0.0087 |

^a^ the area under the emission curve

**Table S3.** Equivalent circuit element values obtained by fitting the impedance spectra for gold (Au), DPPC, DPPC-TS, DPPC-TB (spectra not shown) and DPPC-TSF (Fig. 10A)

| **Surface / Electrolyte** | **Temperature / °C** | **R_Ω_ / Ω cm^2^** | **R_m_ / kΩ cm^2^** | **Z_W_ / kΩ cm^2^** | **CPE_m_ / µF cm^-2^ s^α-1^** | **α_m_** |
| --- | --- | --- | --- | --- | --- | --- |
| **Au** | 30 | 12.47 | 8.41 | 2.26 | 13.96 | 0.890 |
| **DPPC** | 30 | 11 ± 1 | 8.27 | 1.34 | 9.37 | 0.848 |
|  | 32 |  | 9.15 | 1.42 | 10.06 | 0.870 |
|  | 34 |  | 9.46 | 1.28 | 11.81 | 0.873 |
|  | 35 |  | 9.25 | 1.21 | 13.26 | 0.875 |
|  | 36 |  | 8.76 | 1.06 | 15.10 | 0.875 |
|  | 38 |  | 8.17 | 0.97 | 16.28 | 0.880 |
|  | 40 |  | 7.42 | 0.89 | 17.97 | 0.882 |
|  | 42 |  | 6.77 | 0.83 | 19.26 | 0.886 |
|  | 43 |  | 6.28 | 0.79 | 20.31 | 0.890 |
| **DPPC-TS** | 30 | 20 ± 2 | 8.18 | - | 15.33 | 0.857 |
|  | 32 |  | 8.07 | - | 16.39 | 0.854 |
|  | 34 |  | 9.98 | - | 11.82 | 0.870 |
|  | 35 |  | 10.63 | - | 11.71 | 0.871 |
|  | 36 |  | 11.05 | - | 11.37 | 0.872 |
|  | 38 |  | 11.21 | - | 11.09 | 0.874 |
|  | 40 |  | 11.13 | - | 10.88 | 0.874 |
|  | 42 |  | 10.49 | - | 10.53 | 0.877 |
|  | 43 |  | 10.09 | - | 10.48 | 0.878 |
| **DPPC-TSF** | 30 | 17 ± 1 | 7.80 | - | 16.36 | 0.859 |
|  | 32 |  | 8.32 | - | 16.83 | 0.854 |
|  | 34 |  | 9.62 | - | 15.27 | 0.861 |
|  | 35 |  | 9.68 | - | 15.33 | 0.861 |
|  | 36 |  | 11.35 | - | 14.75 | 0.863 |
|  | 38 |  | 12.47 | - | 14.20 | 0.865 |
|  | 40 |  | 12.79 | - | 13.92 | 0.865 |
|  | 42 |  | 12.73 | - | 14.07 | 0.858 |
|  | 43 |  | 12.85 | - | 14.31 | 0.855 |
| **DPPC-TB** | 30 |  | 22.63 | - | 10.75 | 0.886 |
|  | 32 |  | 22.60 | - | 11.04 | 0.885 |
|  | 34 |  | 23.57 | - | 11.06 | 0.884 |
|  | 35 |  | 24.97 | - | 11.02 | 0.882 |
|  | 36 |  | 25.84 | - | 10.86 | 0.882 |
|  | 38 |  | 24.82 | - | 10.59 | 0.884 |
|  | 40 |  | 22.72 | - | 10.28 | 0.887 |
|  | 42 |  | 19.02 | - | 9.93 | 0.890 |
|  | 43 |  | 18.37 | - | 9.81 | 0.890 |

**Table S4.** Band assignment of the ATR-FTIR spectra of pure DPPC and DPPC with TB and TS compound in 25°C and 45°C.

| **DPPC** | | **TB 15 %** | | **TS 15 %** | | **Vibrational band assignment** |
| --- | --- | --- | --- | --- | --- | --- |
| **25°C** | **45°C** | **25°C** | **45°C** | **25°C** | **45°C** |  |
| 3390 | 3390 | 3387 | 3389 | 3386 | 3387 | ν_as_ CH_2_ |
| 2957 | 2957 | 2956 | 2957 | 2956 | 2957 |  |
| 2917 | 2917 | 2917 | 2918 | 2918 | 2917 |  |
| 2850 | 2850 | 2850 | 2850 | 2851 | 2849 | ν_s_ CH_2_ |
| 1735 | 1736 | 1737 | 1736 | 1735 | 1736 | ν C=O |
|  |  | 1708 | 1706 | 1710 | 1706 |  |
| 1486 | 1490 | 1489 | 1490 | 1486 | 1486 | δ_s_ CH_2_ |
| 1467 | 1467 | 1468 | 1466 | 1467 | 1467 |  |
| 1415 | 1416 | 1416 | 1418 | 1416 | 1416 | ω CH_2_ |
| 1376 | 1376 | 1378 | 1378 | 1377 | 1377 |  |
| 1340 | 1340 | 1340 | 1339 | 1358 | 1340 |  |
| 1244 | 1248 | 1245 | 1248 | 1245 | 1248 | ν_as_ PO_2_ |
| 1224 | 1222 | 1221 | 1223 | 1223 | 1222 |  |
| 1149 | 1145 | 1144 | 1137 | 1152 | 1147 | ν CO-O |
| 1090 | 1092 | 1088 | 1090 | 1087 | 1090 | ν C-O-P-O-C |
| 1068 | 1072 | 1068 | 1071 | 1070 | 1074 | ν C-O-PO_2_^-^ |
| 968 | 968 | 969 | 971 | 974 | 971 | ν_as_ N^+^(CH_3_)_3_ |
| **DPPC** | | **TB 5 %** | | **TS 5 %** | | **Vibrational band assignment** |
| **25°C** | **45°C** | **25°C** | **45°C** | **25°C** | **45°C** |  |
| 3390 | 3390 | 3383 | 3384 | 3393 | 3391 | ν_as_ CH_2_ |
| 2957 | 2957 | 2956 | 2956 | 2956 | 2956 |  |
| 2917 | 2917 | 2918 | 2918 | 2917 | 2918 |  |
| 2850 | 2850 | 2850 | 2849 | 2849 | 2850 | ν_s_ CH_2_ |
| 1735 | 1736 | 1733 | 1735 | 1736 | 1736 | ν C=O |
|  |  | 1707 | 1710 | 1705 | 1704 |  |
| 1486 | 1490 | 1490 | 1488 |  | 1487 | δ_s_ CH_2_ |
| 1467 | 1467 | 1468 | 1468 | 1468 | 1467 |  |
| 1415 | 1416 | 1419 | 1418 | 1420 | 1419 | ω CH_2_ |
| 1376 | 1376 | 1377 | 1377 | 1372 | 1372 |  |
| 1340 | 1340 | 1339 | 1339 | 1340 | 1338 |  |
| 1244 | 1248 | 1244 | 1249 | 1245 | 1248 | ν_as_ PO_2_ |
| 1224 | 1222 | 1224 | 1222 | 1220 | 1218 |  |
| 1149 | 1145 | 1143 | 1142 | 1151 | 1140 | ν CO-O |
| 1090 | 1092 | 1088 | 1092 | 1088 | 1091 | ν C-O-P-O-C |
| 1068 | 1072 | 1069 | 1074 | 1069 | 1073 | ν C-O-PO_2_^-^ |
| 968 | 968 | 970 | 971 | 968 | 969 | ν_as_ N^+^(CH_3_)_3_ |

ν – stretching vibrations (ν_s_ -symmetric, ν_as_ - asymmetric), δ_s_ – bending, ω - wagging
